# Subtype-specific neurons from patient iPSCs display distinct neuropathological features of Alzheimer’s disease

**DOI:** 10.1186/s13619-024-00204-y

**Published:** 2024-10-10

**Authors:** Ran Tao, Chunmei Yue, Zhijie Guo, Wenke Guo, Yao Yao, Xianfa Yang, Zhen Shao, Chao Gao, Jianqing Ding, Lu Shen, Shengdi Chen, Naihe Jing

**Affiliations:** 1 Guangzhou National Laboratory, Guangzhou International Bio Island, No. 9 Xing Dao Huan Bei Road, Guangdong Province, 510005 China; 2 Suzhou Yuanzhan Biotechs, Suzhou, 215000 China; 3 CAS Key Laboratory of Computational Biology, Shanghai Institute of Nutrition and Health, Chinese Academy of Sciences, Shanghai, 200031 China; 4 University of Chinese Academy of Sciences, Beijing, 100049 China; 5 XellSmart Biomedical (Suzhou) Co., Ltd, Suzhou, 215000 China; 6 Center for Cell Lineage and Development, CAS Key Laboratory of Regenerative Biology, Guangdong Provincial Key Laboratory of Stem Cell and Regenerative Medicine, New Zealand Joint Laboratory On Biomedicine and Health, Guangzhou Institutes of Biomedicine and Health, GIBH-HKU Guangdong-Hong Kong Stem Cell and Regenerative Medicine Research Centre, Chinese Academy of Sciences, Guangzhou, 510530 China; 7 Department of Neurology & Institute of Neurology, RuiJin Hospital, Shanghai Jiao Tong University School of Medicine, Shanghai, 200020 China; 8 Department of Neurology, Xiangya Hospital, Central South University, Changsha, China; 9 National Clinical Research Center for Geriatric Disorders, Central South University, Changsha, 410028 China; 10 Lab for Translational Research of Neurodegenerative Diseases, Shanghai Institute for Advanced Immunochemical Studies (SIAIS), Shanghai Tech University, Shanghai, 200031 China

**Keywords:** Alzheimer’s disease (AD), iPSC, Cellular model, Basal forebrain cholinergic neuron (BFCN), Cortical glutamatergic neuron

## Abstract

Alzheimer’s disease (AD) is a progressive neurodegenerative disorder characterized by massive neuronal loss in the brain. Both cortical glutamatergic neurons and basal forebrain cholinergic neurons (BFCNs) in the AD brain are selectively vulnerable. The degeneration and dysfunction of these two subtypes of neurons are closely associated with the cognitive decline of AD patients. The determination of cellular and molecular mechanisms involved in AD pathogenesis, especially in the early stage, will largely facilitate the understanding of this disease and the development of proper intervention strategies. However, due to the inaccessibility of living neurons in the brains of patients, it remains unclear how cortical glutamatergic neurons and BFCNs respond to pathological stress in the early stage of AD. In this study, we established in vitro differentiation systems that can efficiently differentiate patient-derived iPSCs into BFCNs. We found that AD-BFCNs secreted less Aβ peptide than cortical glutamatergic neurons did, even though the Aβ42/Aβ40 ratio was comparable to that of cortical glutamatergic neurons. To further mimic the neurotoxic niche in AD brain, we treated iPSC-derived neurons with Aβ42 oligomer (AβO). BFCNs are less sensitive to AβO induced tau phosphorylation and expression than cortical glutamatergic neurons. However, AβO could trigger apoptosis in both AD-cortical glutamatergic neurons and AD-BFCNs. In addition, AD iPSC-derived BFCNs and cortical glutamatergic neurons exhibited distinct electrophysiological firing patterns and elicited different responses to AβO treatment. These observations revealed that subtype-specific neurons display distinct neuropathological changes during the progression of AD, which might help to understand AD pathogenesis at the cellular level.

**Supplementary Information:**

The online version contains supplementary material available at 10.1186/s13619-024-00204-y.

## Background

Alzheimer’s disease (AD), characterized by progressive impairment of memory and cognition, is one of the most prevalent neurodegenerative disorders. Both sporadic AD (SAD) and familial AD (FAD) share common pathological features, including the accumulation of β-amyloid peptide (Aβ) and hyperphosphorylated tau, as well as cerebral neuronal loss (Jack and Holtzman 2013). However, because of the inaccessibility to the living neurons of patients, the cellular changes at the early stages of AD remain elusive.

Previous studies using postmortem samples revealed that, in the process of AD pathology, the origin and spreading of Aβ deposits or tau inclusions showed distinct patterns (Braak and Braak 1991, 1997; Geula et al. 2021; Goedert 2015; Thal et al. 2002). Aβ plaques were first observed in the basal temporal and orbitofrontal neocortex and then in almost the entire neocortex, followed by the hippocampus, amygdala, diencephalon and basal ganglia. However, neurofibrillary tangles containing hyperphosphorylated tau followed a disparate spreading direction, as they developed initially in the locus coeruleus and entorhinal cortex, gradually spread to the hippocampus and then to large parts of the neocortex. These findings suggested that different cerebral regions, or even subgroups of neurons, may exhibit specific pathological features during the process of AD. To obtain a comprehensive view of AD pathology, further investigations on different neuronal subtypes of the patient’s brain are necessary.

Both cortical glutamatergic neurons and basal forebrain cholinergic neurons (BFCNs) are selectively vulnerable in AD brain. However, as BFCNs are difficult to be induced from human pluripotent stem cells in vitro, few studies on the role of BFCNs in the early pathogenesis of AD have been reported. BFCNs provide the major cholinergic projection to the cerebral cortex and hippocampus, and critically participate in cognitive functions, including learning, memory and attention. Up to 95% loss of choline acetyltransferase (ChAT) activity and 90% loss of acetylcholinesterase (AChE) activity were found in the cerebral cortex of AD patients, which indicates widespread depletion of cholinergic innervation in AD brains (Geula et al. 2021). Although the effects seemed to be transient, therapies based on cholinesterase or choline acetyltransferase inhibitors to reverse cholinergic hypofunction have consistently improved the cognitive abilities of AD patients, which further confirmed the “cholinergic hypothesis” in Alzheimer’s disease (Geula et al. 2021; Holtzman et al. 2011; Martinez et al. 2021). Nevertheless, inadequate knowledge of BFCN precluded further application in the treatment of AD.

Human somatic cell-derived induced pluripotent stem cells (iPSCs) carrying patient-specific genetic information can be induced into functionally specialized neuronal subtypes, and the in vivo pathological process can be mimicked in vitro. In our previous study, we established an efficient protocol to direct human iPSCs to differentiate into cortical glutamatergic neurons and generated a cellular model of AD (Tao et al. 2020). However, BFCNs are difficult to be induced from human iPSCs in vitro. To date, only limited studies have reported successful acquisition of BFCNs from human iPSCs and from Alzheimer’s disease models (Martinez et al. 2021). These studies transdifferentiate skin fibroblasts into BFCNs with virus-mediated expression of transcription factors (Ma et al. 2020), combined neural differentiation with the overexpression of BFCN-associated transcription factors and enrichment of BFCN progenitors via FACS (Duan et al. 2014) or cocultured these cells with astrocytes to increase differentiation efficiency (Hu et al. 2016).

Here, we first established a new protocol to induce iPSCs to differentiate into BFCNs using only small molecules and growth factors. Then, we measured the pathological features of AD patient-specific BFCNs and cortical glutamatergic neurons. Compared with AD-iPSC-induced cortical glutamatergic neurons, BFCNs secrete fewer Aβ peptides and are less sensitive to the effects of Aβ42 oligomers (AβO) on tau phosphorylation and expression. Nevertheless, both cortical glutamatergic neurons and BFCNs were induced to undergo apoptosis upon AβO treatment. Furthermore, BFCNs and cortical glutamatergic neurons exhibit different electrophysiological firing patterns and responses to toxic AβO. Taken together, these findings reveal novel pathological features of AD in patient iPSC-derived BFCNs, which might help to further describe AD pathology at the cellular level and hopefully facilitate efforts to develop new strategies for the treatment of Alzheimer’s disease.

## Results

### Establishment of an efficient differentiation protocol from AD patient-specific iPSCs to BFCNs

FAD and SAD patient-specific iPSCs were generated from peripheral blood mononuclear cells (PBMNCs). In somatic cell reprogramming, nonintegrative episomal vector-mediated nucleofection was used to maintain the integrity of genetic information (Chou et al. 2011; Dowey et al. 2012). The donor information is summarized in Supplementary Table S1. The iPSCs generated from control-2 and control-3 cells have been used previously (Tao et al. 2020).

**Fig. 1:**
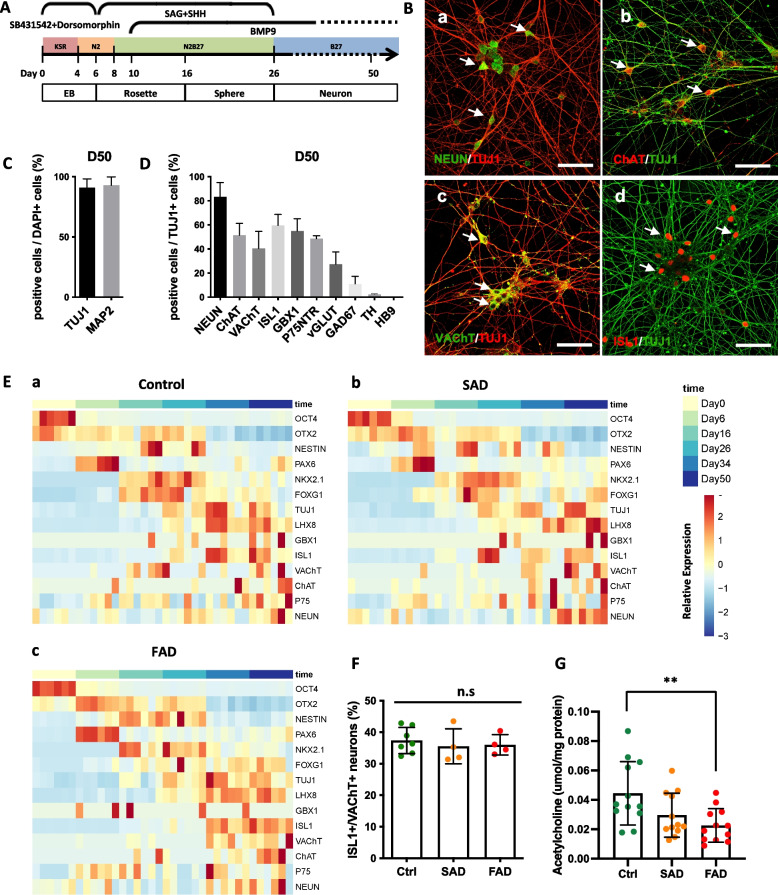
Induction of human iPSC-derived basal forebrain cholinergic neurons. **A** Schematic representation of the modified protocol used to direct the differentiation of human iPSCs to basal forebrain cholinergic neurons. **B** Immunofluorescence images of human iPSC-derived cells expressing the mature neuronal marker NEUN (a), the cholinergic neuronal marker ChAT (b), VAChT (c) and ISL1 (d) at differentiation day 50. Arrows, representative neurons with positive staining. Scale bars, 50 μm. **C** The quantification of the percentage of neurons induced from human iPSCs is shown in Fig. S1A-a and b. *n* = 3. The data are presented as the means ± SDs. **D** The percentages of different neuronal subtypes are shown in Fig. 1B and S1A. *n* = 3. The data are presented as the means ± SDs. **E** Heatmap of marker gene expression by RNA-seq during the neural differentiation of control, SAD and FAD patient-specific iPSCs into BFCNs. **F** Percentage of ISL1^+^/VAChT.^+^ neurons derived from control, SAD and FAD patient-specific iPSC lines. **G** Acetylcholine secreted by control, SAD and FAD patient-specific iPSC-derived BFCNs. Quantity of acetylcholine secreted into culture medium was normalized to the quantity of total protein in the whole-cell lysates. The data are presented as means ± SD. **P < 0.01

To study AD-specific molecular changes in living BFCNs, we first established a differentiation protocol based on the current understanding of the developmental process in vivo, enabling the derivation of BFCNs from human iPSCs. As shown in Fig. 1A, only chemically defined factors were used with optimized durations and concentrations in this procedure. High dose of ventralizing morphogen sonic hedgehog (SHH) and its activator smoothened agonist (SAG) induced the ventralization of neural stem cells (NSCs)/neural progenitor cells (NPCs) (Campbell 2003; Marin and Rubenstein 2001). BMP9 is an important cholinergic differentiation factor that induce and maintain the cholinergic phenotype of BFCN by directly inducing the expression of BFCN specific genes and up-regulated acetylcholine synthesis (Lopez-Coviella et al. 2000, 2005; Schnitzler et al. 2010).

Following this protocol, human iPSCs were induced to differentiate into mature neurons within 50 days according to the expression of the neuronal marker genes TUJ1 and MAP2 (Fig. S1a-b, Fig. 1C) and the mature neuronal marker NEUN (Fig. 1Ba, D). The majority of the neurons derived from human iPSCs expressed the BFCN-specific markers ChAT, VAChT, ISL1, GBX1 and P75NTR (Fig. 1B b-d, D, Fig. S1 Ac-d). A small number of vGLUT^+^ glutamatergic neurons (28.1%), GAD67^+^ GABAergic neurons (11.5%) and TH^+^ dopaminergic neurons (2.3%) were found among the induced neurons (Fig. S1A e-g, Fig. 1D). However, the spinal cord motor neuron (another cholinergic neuron in the central nervous system)-specific marker HB9 could not be detected in the neurons induced by this protocol (Fig. S1A h, Fig. 1D). At day 55~65, whole-cell patch-clamp recording was performed on randomly picked neurons. Most of the iPSC-derived neurons were able to fire action potentials (APs) (9/12), especially repetitive APs (8/12) (Fig. S1B). Spontaneous APs (Fig. S1C) or post-synaptic currents (PSCs) (Fig. S1D) were also identified in some induce neurons. These results indicated that, iPSC derived neurons expressed membrane properties and were functionally mature. Thus, following the optimized differentiation protocol that only used small molecules and growth factors, we efficiently induced human iPSCs to differentiate into BFCNs.

Next, we applied both SAD and FAD patient-specific iPSCs to the established BFCN differentiation system. As expected, iPSCs derived from SAD and FAD patients could be differentiated into BFCNs with similar molecular trajectories as those of control iPSCs, as evidenced by the expression patterns of representative marker genes involved in BFCN development by RNA sequencing (Fig. 1E). SAD, FAD and control iPSCs differentiated into BFCNs with comparable efficiency (Fig. 1F). In addition, the ability to release acetylcholine by iPSC-induced BFCNs were measured. Acetylcholine secretion could be detected in the cultures of both healthy control and AD patient specific BFCNs. However, FAD patient-specific BFCNs secreted significantly lower acetylcholine levels than healthy control (Fig. 1G).

Overall, we established an in vitro differentiation system that can efficiently induce the generation of BFCNs from control, SAD and FAD patient-derived iPSCs. Patient-specific iPSC-induced BFCNs may further be used to model Alzheimer’s disease in vitro*.*

### AD patient-specific BFCNs secrete less Aβ than cortical glutamatergic neurons

To study the neuronal subtype-specific pathological characteristics, we differentiated iPSCs into cortical glutamatergic neurons (referred to as cortical neurons hereinafter) and BFCNs, respectively. For each individual, 2 or 3 iPSC lines generated from single clone were utilized for parallel experiments to systematically evaluate the emergence of AD-related pathology. Cortical neurons were differentiated from both control and AD patient-specific iPSCs following a previously published protocol (Tao et al. 2020, Materials and methods). Human iPSC-derived cortical neurons expressed cortical marker TBR1, mature neuronal marker NEUN and glutamatergic neuronal marker vGLUT at day 50 after differentiation (Fig. S1E), which indicated that they were mature cortical glutamatergic neurons. About 92% of induced neurons expressed vGLUT, while the ratio was not significantly altered among healthy control, SAD and FAD group (Fig. S1F).

**Fig. 2:**
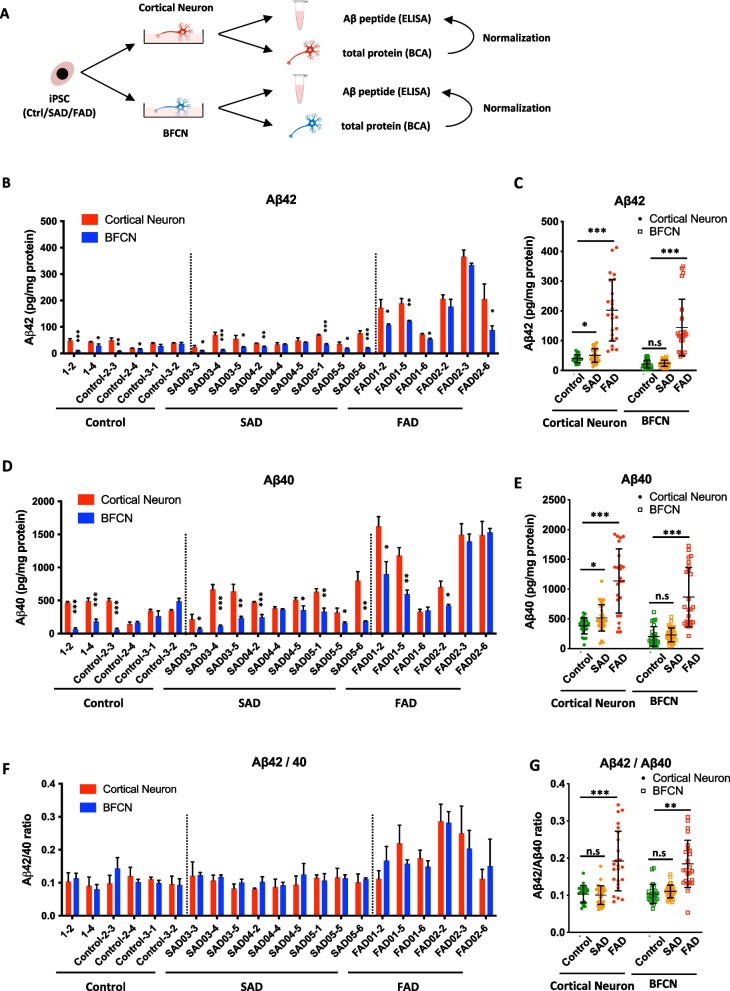
BFCNs secrete less Aβ than cortical neurons. **A** Schematic of the experimental design for the sample collection and quantification of Aβ peptides secreted by iPSC induced cortical neurons and BFCNs. **B** ELISA was used to measure the levels of Aβ42 secreted by cortical neurons or BFCNs derived from the different iPS cell lines. *n* = 4. **C** Comparison of the volume of Aβ42 peptide secreted by control, SAD and FAD-iPSC-derived cortical neurons and BFCNs. **D** ELISA was used to measure the levels of Aβ40 secreted by cortical neurons or BFCNs derived from the different iPS cell lines. *n* = 4. **E** Comparison of the volume of Aβ40 peptide secreted by control, SAD and FAD-iPSC-derived cortical neurons and BFCNs. **F** ELISA was used to measure the ratio of Aβ42 to Aβ40 secreted by cortical neurons or BFCNs derived from the different iPS cell lines. *n* = 4. **G** Comparison of the ratio of Aβ42 to Aβ40 peptide secreted by control, SAD and FAD-iPSC-derived cortical neurons and BFCNs. The concentration of Aβ peptides in the culture medium was normalized to the total protein concentration in the whole-cell lysates. Data are presented as the means ± SDs. *P < 0.05, **P < 0.01 and ***P < 0.001

The levels of secreted Aβ peptides from these two distinct neuronal subtypes were measured via ELISA of the Aβ peptides in the cell culture medium on the 50th day of differentiation (Fig. 2A). Interestingly, we found that most iPSC-induced cortical neurons secreted more Aβ42 and Aβ40 than BFCNs that differentiated from the same iPS cell line (Fig. 2B, D). Even though the secretion of Aβ varies among different individuals, both FAD patient-specific cortical neurons and BFCNs secreted markedly greater amounts of Aβ than SADs and controls (Fig. 2C, E). However, for SAD iPSC-derived neurons, the average levels of Aβ42 and Aβ40 secreted by cortical neurons were significantly elevated, while the amount of Aβs secreted by BFCNs was comparable to that secreted by controls (Fig. 2C, E). These results indicate that the capacity for Aβ secretion differs between BFCNs and cortical neurons. By calculating the ratio of secreted Aβ42 to Aβ40 from both neuronal subtypes, we found that the Aβ42/Aβ40 ratios were comparable (approximately 0.1) between the control and SAD groups, whereas FAD patient-specific neurons harboring the PSEN1 I202F (c.A604T) or APP V717I (c.G2149A) mutation showed significantly increased Aβ42/Aβ40 ratios in both cortical neurons and BFCNs, which was consistent with the in vivo Aβ production in AD patients (Fig. 2F, G).

To explain the difference between cortical neurons and BFCNs in Aβ secretion, we further analyzed the expression of the key factors involved in Aβ generation, including the amyloid precursor protein (APP), α-secretase ADAM10 and β-secretase BACE1 in cortical neuron and BFCNs. Compared with cortical neurons, the expression of APP was reduced in BFCNs, especially in AD patient derived BFCNs, while the repression of BACE1 and ADAM10 was not significantly altered (Fig. S2). The disparate APP expression might result in the difference in Aβ secretion in cortical neurons and BFCNs.

Taken together, the established in vitro differentiation system for both cortical neurons and BFCNs successfully mimics the AD pathology of Aβ42 and Aβ40 production. Moreover, compared with cortical neurons, BFCNs produced less Aβ42 and Aβ40, which suggests that subtype-specific neurons exhibit distinct features related to Aβ secretion.

### Tau phosphorylation responded differently to toxic AβO between cortical neurons and BFCNs

Soluble Aβ42 oligomers (AβOs) are considered to be the main toxic form of Aβ peptides. Their interactions with receptors on cell membranes may induce multiple downstream cascade reactions, including tauopathy and neuronal death (Canter et al. 2016; El-Agnaf et al. 2000; Goedert 2015; Hardy and Selkoe 2002; Hardy and Higgins 1992). To test the responsiveness of cortical neurons and BFCNs to toxic AβO, the in vitro synthesized human Aβ42 (Aβ_1-42_) oligomers were supplemented into neuronal cultures, and the peptides with the inverted amino acid sequence of human Aβ42 (Aβ_42-1_) were used as controls.

**Fig. 3:**
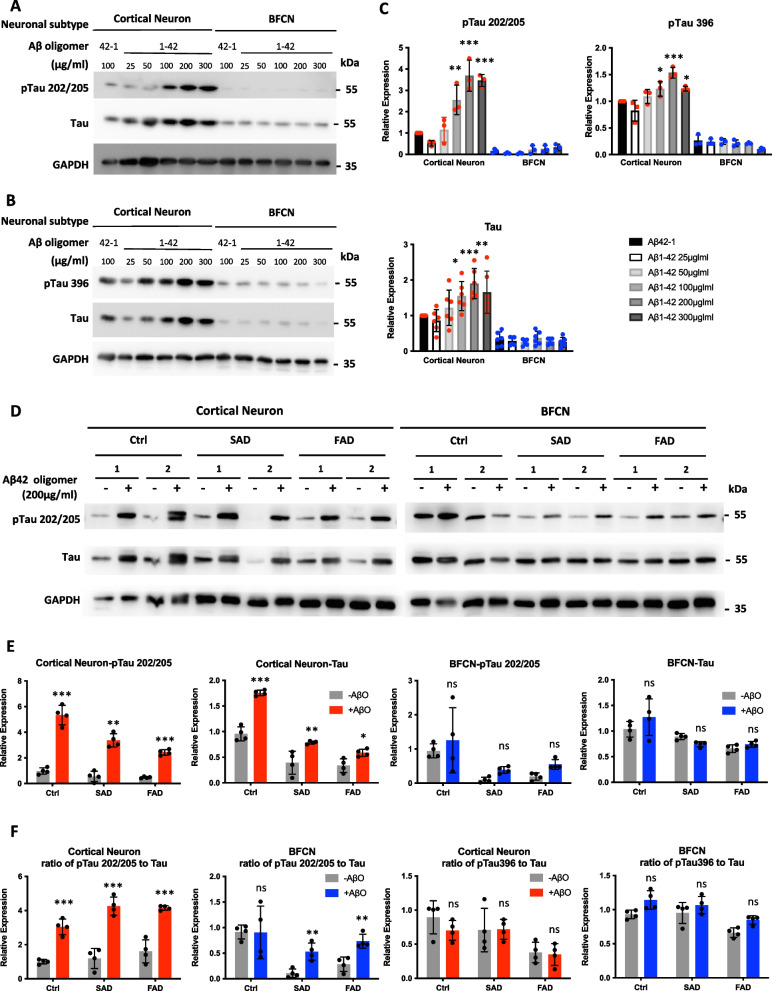
Cortical neurons and BFCNs exhibit different responses to toxic AβO via tau phosphorylation. **A**, **B** Control iPSC-derived cortical neurons and BFCNs on differentiation day 48 were treated with synthesized human Aβ1-42 oligomers at different concentrations ranging from 25 to 300 μg/ml. After 48 h, the levels of phosphorylated tau (A. pTau 202/205, B. pTau396) and total tau were analyzed via western blotting. Human Aβ42-1 oligomers were used as scramble negative controls. **C** The levels of the related proteins were quantified via western blotting, as shown in Fig. 3A, B. The data are presented as the means ± SDs. *P < 0.05, **P < 0.01 and ***P < 0.001. **D** Control-, SAD- and FAD-iPSC-derived cortical neurons and BFCNs were treated with synthesized human Aβ1-42 (Aβ42) oligomers at a concentration of 200 μg/ml. After 48 h, the levels of pTau202/205 and total tau were analyzed via western blotting. **E** The levels of the proteins analyzed by western blot shown in Fig. 3D. The data are presented as the means ± SDs. *P < 0.05, **P < 0.01 and ***P < 0.001. **F** Ratio of pTau to total Tau protien levels in cortical neurons or BFCNs shown in Fig. 3D and supplementary Fig. S3C. Data are presented as the means ± SDs. *P < 0.05, **P < 0.01 and ***P < 0.001

To determine the optimal conditions for the addition of Aβ to in vitro cultured neurons, we first treated healthy control iPSC-derived cortical neurons and BFCNs with freshly prepared AβO at different concentrations at day 48 after differentiation. Then the phosphorylation of tau at Ser202/Thr205 (pTau202/205), Ser396 (pTau396) or Thr231 (pTau231), as well as total tau expression, was measured via western blotting at 48 h after treatment (Fig. 3A-C, Fig. S3A, B). Both the phosphorylation and expression of tau in cortical neurons were induced by AβO in a dose-dependent manner, and tau phosphorylation peaked at 200 μg/ml AβO when detected with all three antibodies. In contrast, the phosphorylation and expression levels of tau in BFCNs were scarcely affected by AβO treatment (Fig. 3A-C, Fig. S3A, B).

Next, Tau phosphorylation at Ser202/Thr205 and Ser396 in response to 200 μg/ml AβO was further measured in SAD and FAD iPSC-induced neurons at day 50 after differentiation. As in healthy controls, pTau 202/205 and total tau protein levels, as well as their ratio were significantly induced by AβO in SAD- and FAD-induced cortical neurons (Fig. 3D-F). However, although the phosphorylation at Ser396 was elevated by AβO, their ratio to total Tau was not altered (Fig. S3C, D, Fig. 3F). For both control and AD specific BFCNs, the expression and phosphorylation of tau in BFCNs was not induced by AβO (slightly increased in SAD- and FAD-BFCNs, without significance). However, the ratio of pTau 202/205 to Tau was significantly elevated in SAD- and FAD-BFCNs.

These results suggest that AβO can induce the phosphorylation and expression of tau in cortical neurons, but BFCNs (especially non-AD BFCNs) exhibit less sensitivity to toxic AβO in terms of tau phosphorylation and expression.

### Toxic AβO induced apoptosis in both cortical neurons and BFCNs

**Fig. 4:**
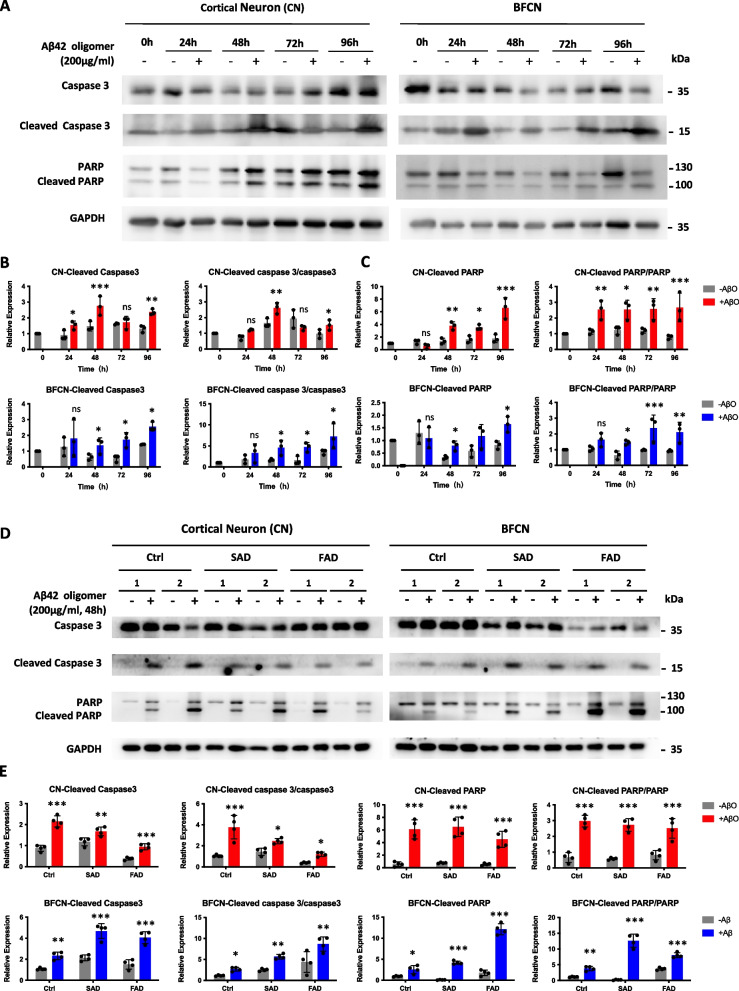
Toxic AβO induced the activation of the caspase 3 pathway in both cortical neurons and BFCNs. **A** Control iPSC-derived cortical neurons and BFCNs were treated with synthesized human Aβ42 oligomers (200 μg/ml) for different durations ranging from 24 to 96 h. On differentiation day 50, the levels of full-length and cleaved caspase 3, as well as its downstream target PARP, were analyzed via western blotting. **B** The quantification of cleaved caspase 3 protein levels and their ratios to full-length caspase 3 protein levels was performed via western blotting as shown in Fig. 4A. The data are presented as the means ± SDs. *P < 0.05, **P < 0.01 and ***P < 0.001. **C** The quantification of cleaved PARP protein levels and their ratios to full-length PARP protein levels was performed via western blotting as shown in Fig. 4A. The data are presented as the means ± SDs. *P < 0.05, **P < 0.01 and ***P < 0.001. **D** Control-, SAD- and FAD-iPSC-derived cortical neurons and BFCNs were treated with synthesized human Aβ42 oligomers (200 μg/ml) for 48 h. On differentiation day 50, the levels of full-length and cleaved caspase 3, as well as its downstream target PARP, were analyzed via western blotting. **E** The quantification of the related protein levels and their ratios was performed via western blotting, as shown in Fig. 4C. The data are presented as the means ± SDs. *P < 0.05, **P < 0.01 and ***P < 0.001

In addition to Aβ production and tau phosphorylation, neuronal loss in multiple brain regions is one of the most common and important features of Alzheimer’s disease (Goel et al. 2022). To study apoptosis in the AD-like neurotoxic niche, in vitro-differentiated cortical neurons and BFCNs were treated with 200 μg/ml of freshly synthesized AβO, after which the occurrence of apoptotic features was tested at day 50 after differentiation. First, we treated control iPSC-derived neurons with AβO to determine the duration required for apoptosis induction. In both cortical neurons and BFCNs, the level of cleaved caspase 3 (the activated form of caspase 3) began to increase as early as 24 h after administration, indicating the activation of the apoptosis-associated signaling pathway (Fig. 4A, B). After 48 h of treatment, the activation of caspase 3 was significantly induced in both neuronal subtypes (Fig. 4A, B). Moreover, the cleavage of PARP, downstream of activated caspase 3, was elevated since 48 h after AβO treatment in both cortical neurons and BFCNs (Fig. 4A, C). These results suggest that the apoptosis pathway was obviously activated in both neuronal subtypes derived from control iPSCs after AβO treatment for 48 h. Next, we measured the activation of the caspase 3 pathway in both SAD and FAD patient-derived cortical neurons and BFCNs. We found that 48 h of AβO treatment could efficiently induce cellular apoptosis in both cortical neurons and BFCNs (Fig. 4D, E).

**Fig. 5:**
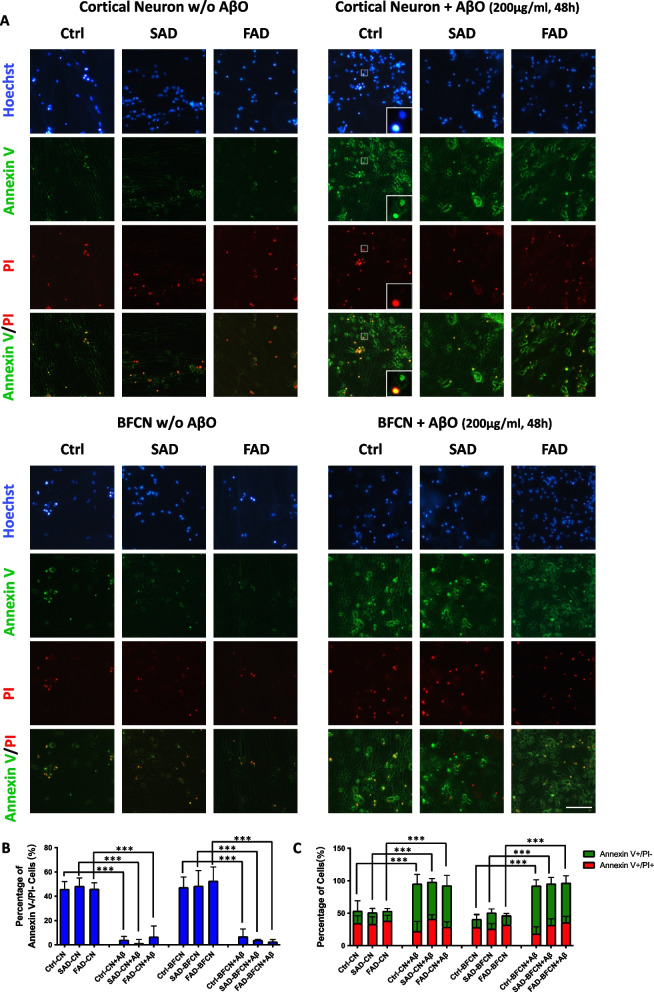
Toxic AβO induced the apoptosis of both cortical neurons and BFCNs. **A** Annexin V/PI staining was performed on iPSC-induced cortical neurons and BFCNs with or without AβO treatment on differentiation day 50. Magnified views of boxes are shown as an example of positive cells. Scale bars, 100 μm. **B**, **C** The percentages of Annexin V-/PI- normal neurons (B), early apoptotic Annexin V + /PI- cells (C-green) and Annexin V + /PI + dead cells (C-red) in each group were quantified. The data are presented as the means ± SDs. *P < 0.05, **P < 0.01 and ***P < 0.001

The externalization of phosphatidylserine occurs early in neuronal apoptosis and can be labelled with fluorescent annexin V, and live and early apoptotic cells with intact membranes are resistant to propidium iodide (PI) penetration (Rimon et al. 1997). Thus, we used annexin V staining as a marker of early apoptotic neuronal cells and PI as a marker of late apoptotic and dead cells. As shown in Fig. 5A (left panels), without AβO treatment, approximately 50% of the cortical neurons and BFCNs derived from control, SAD and FAD iPSCs were healthy (Annexin V-/PI-, blue bars in Fig. 5B). Whereas the percentage of normal cells (Annexin V-/PI-) was greatly reduced to less than 10% (Fig. 5A-right panels and B) after AβO treatment for 48 h. At the same time, the proportion of early apoptotic neurons (Annexin V + /PI-, green bars in Fig. 5C) increased dramatically from approximately 15% to 50%~60% in both cortical neurons and BFCNs (Fig. 5A, C). However, the percentage of dead neuronal cells (Annexin V + /PI + , red bars in Fig. 5C) did not change much after 48 h of AβO treatment (Fig. 5A, C). These results indicate that AβO could induce similar apoptotic effects on both cortical neurons and BFCNs.

### AD-specific cortical neurons and BFCNs exhibit typical electrophysiological features

**Fig. 6:**
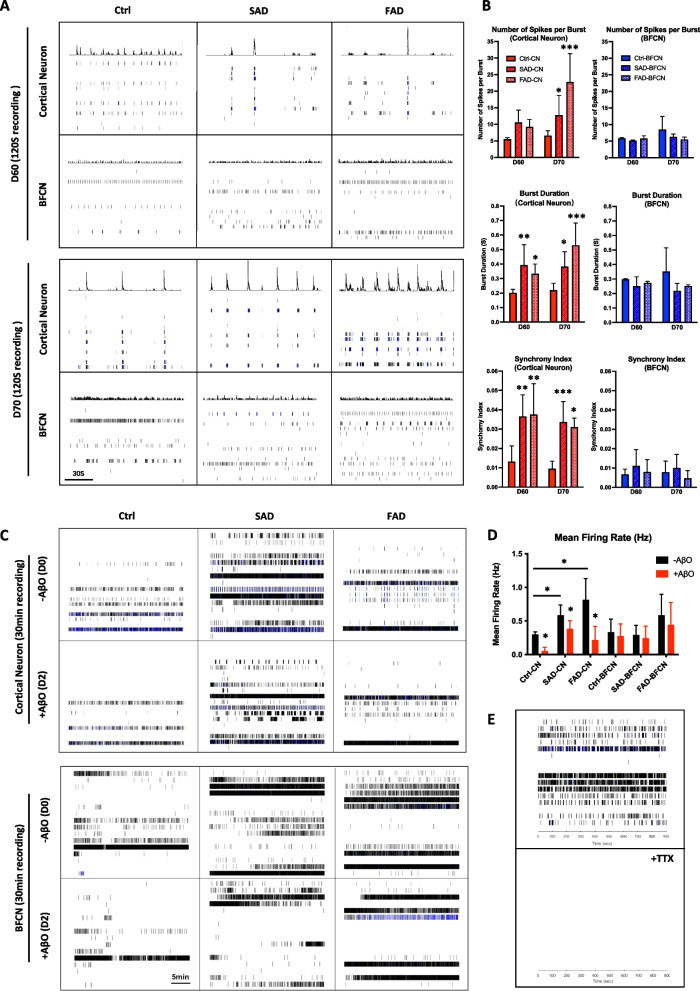
MEA recording revealed the electrophysiological features associated with neural subtypes in AD-iPSC-derived cortical neurons and BFCNs. **A** Example raster plots of spikes showing synaptic events from a 120 s recording on day 60 or 70 after neural differentiation of iPSCs. Peak maps representing the overall spiking events over time in each well are shown above each raster plot. **B** Quantification of MEA parameters: number of spikes per burst (top), burst duration (middle) and synchrony index (bottom). The data are presented as the means ± SDs. *P < 0.05, **P < 0.01 and ***P < 0.001. **C** Raster plots showing the spiking events recorded by the MEA system at 1800s before or 2 days after AβO treatment. **D** Quantification of the mean firing rate of cortical neurons or BFCNs in the control, SAD and FAD groups indicated changes before or after AβO treatment. The data are presented as the means ± SDs. *P < 0.05. **E** Raster plots showing the blockage of electrical activity by tetrodotoxin (TTX) in the MEA system

To further assess the functions of patient-specific neurons, we used the super high-resolution multielectrode array (MEA) system to record the spontaneous electrical activity of iPSC-derived cortical neurons and BFCNs. Spiking events recorded in 120 s were represented with peak maps and raster plots (Fig. 6A). On day 60 after iPSC differentiation, both SAD- and FAD-derived cortical neurons began to exhibit firing-pausing-firing spontaneous action potentials. Concurrently, BFCNs maintain continuous spiking patterns, similar to those of control cortical neurons (Fig. 6A-upper panel, B). Bursting was defined as intermittent periods of high-frequency firing and was recognized to be associated with a range of physiological processes of functional neuronal networks (Cotterill and Eglen 2019). Compared with those of control cortical neurons, the number of spikes per burst was significantly elevated in both SAD and FAD iPS-derived cortical neurons at differentiation day 70, while there was no change in BFCNs (Fig. 6A-lower panel). Moreover, synchronous bursting was observed in SAD- and FAD-specific cortical neurons on day 60, which suggested that an early mature neuronal network had developed. On day 70, network bursting was fired regularly by SAD and FAD cortical neurons and occasionally in control cortical neurons (Fig. 6A). However, synchronized bursting could hardly be observed in BFCNs (Fig. 6A). In addition, SAD and FAD iPS-induced cortical neurons exhibited increased burst duration and synchrony index than BFCNs and control cortical neurons since day 60, and these changes were maintained thereafter (Fig. 6B).

Next, the effects of neuronal subtype-specific responses to toxic AβO on electrical activity were further evaluated. With 30 min recording, compared with those of the control group, the SAD- and FAD-derived cortical neurons exhibited greater electrical activity, as the mean firing rate was significantly elevated on day 80 after differentiation (D0, -AβO) (Fig. 6C, D). However, the mean firing rate of the BFCNs from SAD and FAD patients did not significantly differ from that of the control BFCNs on day 80 (Fig. 6C, D). After AβO treatment for 48 h (D2, + AβO), the mean firing rate decreased markedly in cortical neurons derived from the control, SAD and FAD groups (Fig. 6C, D). For the BFCNs derived from the control, SDA and FAD groups, AβO treatment did not significantly reduce the mean firing rate (Fig. 6C, D). Extracellular voltage traces also confirmed that the spontaneous electrical activity of cortical neurons decreased in response to AβO treatment, but this activity did not change much in BFCNs (Fig. S4). Spikes can be fully blocked by the voltage-gated sodium channel blocker tetrodotoxin (TTX), indicating the presence of functional sodium channels involved in electrical activities recognized by the MEA system (Fig. 6E).

Taken together, differences in the amount and pattern of spike and burst firing were observed between iPSC-derived cortical neurons and BFCNs. AD patient-specific cortical neurons seemed to perform an early maturation of neuronal network. In addition, the electrical activities of cortical neurons were suppressed by acute AβO treatment, while those of BFCNs were largely insensitive to extracellular AβO treatment.

## Discussion

In this study, we established a new protocol to induce BFCNs from human iPSCs with small molecules and growth factors, and compared the pathological features of AD patient iPSC-derived BFCNs and cortical glutamatergic neurons. We found that BFCNs secreted less Aβ than cortical neurons. In addition, although BFCNs were less sensitive to AβO in tau phosphorylation and expression, both cortical neurons and BFCNs were similarly induced to undergo apoptosis upon AβO treatment. Furthermore, AD-BFCNs and AD-cortical neurons exhibited distinct electrophysiological features and exhibited different responses to AβO treatment. These results suggested that different subtypes of neurons derived from AD patient-specific iPSCs displayed distinct neuropathological changes, which might be helpful in further understanding of the cell type-specific features of AD pathology.

Compared with human ESCs, human iPSCs showed significantly reduced efficiency and increased variability in neural differentiation capacity (Chambers et al. 2012; Hu et al. 2010; Kim et al. 2010). To date, most iPSC-based works on AD modeling have focused mainly on cortical neurons, as they are massively lost in the AD brain and easily to be induced in vitro. The BFCN is one of the most notable subgroups of cells that are selectively vulnerable in Alzheimer’s disease (Auld et al. 2002; Mesulam 2004; Mufson et al. 2003). However, it was difficult to be induced from human iPSCs. Only a handful of studies have reported successfully differentiating human iPSCs into BFCNs to model Alzheimer’s disease (Martinez et al. 2021). These works used transient expression of BFCN-associated transcription factors, enrichment of BFCN progenitors via FACS, or coculture with astrocytes to increase differentiation efficiency (Duan et al. 2014; Hu et al. 2016). Here, based on a comprehensive understanding of the developmental process of BFCNs in vivo, we established a differentiation protocol to derive mature BFCNs from iPSCs with small molecules and growth factors only (Fig. 1 A-D, Fig. S1A-D).

According to postmortem studies on the brains of AD patients, Aβ plaques and tau tangles were found to initiate at different brain regions and spread through distinct directions (Braak and Braak 1991, 1997; Geula et al. 2021; Goedert 2015; Thal et al. 2002). These findings suggested that different cerebral neuron subgroups may exhibit different phenotypes during the pathogenesis of AD. Patient-iPSC derived neurons with specific subtypes have been verified to be able to mimic the cell type-associated abnormalities in vivo. In the brains of AD patients, Aβ plaques always existed in the neocortex, limbic and paralimbic regions, while appeared in brainstem and cerebellum only in a few severe cases. Consistently, it was reported that FAD-iPSC derived rostral neurons showed increased Aβ generation and tau responses to Aβ, in addition, they are more sensitive than caudal neurons (Muratore et al. 2017). These results further confirmed that AD associated phenotypes are specific for different neuronal subtypes.

Familial AD patients carry dominant mutations in APP, PSEN1or PSEN2 genes, which directly affected the generation of Aβs. No gene or mutation has been reported to cause SAD directly. However, by twin studies, the heritability for SAD was estimated to be as high as 58% to 79% (Gatz et al. 2006). According to genome-wide association studies (GWAS), a range of risk genes or loci have been detected to be associated with the development of SAD, including APOE, BIN1, PICALM etc. (Avramopoulos 2009; Karch et al. 2014; Wang et al. 2016). In this study, AD-associated phenotypes, including increased Aβ secretion and altered electrical activities, were found in multiple SAD-patient specific cortical neurons. It might by induced by the expression of AD-associated risk factors. However, in this study, we only concluded the common phenotypes in SAD-specific cortical neurons and BFCNs. To explore the functions of specific risk factor, further studies for individual SAD patient carrying specific genetic information should be performed.

Both cortical glutamatergic neurons and BFCNs are strongly affected in the AD brain. However, a systematic comparison of the neuropathological features of BFCNs and cortical neurons has not been described yet. By comparing cortical neurons derived from both normal and AD iPSCs, we found that BFCNs secreted less Aβ42 and Aβ40 (Fig. 2B-E). The average Aβ concentration secreted by SAD-derived cortical neurons was significantly greater than that secreted by healthy controls (Aβ42: *p* = 0.04281, Aβ40: *p* = 0.01027). However, the levels of Aβ42 and Aβ40 secreted by SAD-derived BFCNs were comparable to those secreted by control BFCNs (Aβ42: *p* = 0.33059, Aβ40: *p* = 0.48706). For FAD patient-specific cortical neurons and BFCNs, the average secretion level of Aβs was much greater than that of control and SADs (Fig. 2B-E). This finding was consistent with the increase in Aβ secretion by BFCNs derived from AD patient-specific iPSCs with the PS2^N141I^ mutation (Duan et al. 2014; Ortiz-Virumbrales et al. 2017), suggesting that different neuronal subtypes might have distinct neuropathological features during the initiation of Alzheimer’s disease.

Following the neural induction protocol in this study, majority of the neurons that derived from human iPSCs were BFCNs. Meanwhile, a small proportion of glutamatergic neurons (28.1%), GABAnergic neurons (11.5%) and dopaminergic neurons (2.3%) were also induced (Fig. 1B, D, Fig. S1A). Cortical glutamatergic neurons were induced from human iPSCs following a previously published protocol. Glutamatergic neurons (89.3%), as well as GABAergic neurons (6.9%) and dopaminergic neurons (5.1%) were induced from iPSCs (Tao et al. 2020). In both differentiation systems, glutamatergic and cholinergic neurons were dominant, while the proportion of GABAergic and dopaminergic neurons was very low and comparable. In this study, although most of the main assays were performed in bulk, the impact of non-cholinergic neurons on conclusions was probably negligible.

The amyloid cascade hypothesis proposed that Aβ is the original trigger of a cascade of alternations in the AD brain and may exert its effect through inducing tau hyperphosphorylation and forming neurofibrillary tangles (Hardy and Selkoe 2002; Hardy and Higgins 1992). In this study, we confirmed that both tau phosphorylation and neuronal apoptosis could be induced by AβO in cortical neurons. However, BFCNs were less sensitive to toxic AβO in tau phosphorylation and expression than cortical neurons (Fig. 3, Fig. S3). Interestingly, although cortical neurons and BFCNs showed differential responses to toxic AβO via tau phosphorylation, apoptosis was induced similarly in both neuronal subtypes (Fig. 4, 5). It suggested that there might be some differences in the pathological process between cortical neurons and BFCNs. Apoptosis in BFCNs might be induced by acute high-dose AβO exposure through a tau-independent pathway in early AD pathology.

In addition, the BFCNs and cortical neurons exhibited different electrophysiological properties. SAD and FAD patient-specific cortical neurons expressed firing-pausing-firing spontaneous action potentials with increased firing properties and network synchrony (Fig. 6A, B), which was consistent with the neuronal hyperexcitability, epilepsy and hypersynchrony observed in early AD (Balusu et al. 2023; Busche et al. 2008; Palop and Mucke 2016; Shah et al. 2022). However, similar phenotypes were not detected in AD BFCNs on day 70 of neural differentiation (Fig. 6A, B). With AβO treatment, electrophysiological deficits were significantly induced in cortical neurons, while those in BFCNs were only slightly altered (Fig. 6C, D, Fig. S4). Tau was reported to be involved in the modulation of sensitivity to excitotoxins and may be involved in the regulation of neuronal activity (Roberson et al. 2007). The hyperexcitability of neurons, as well as behavioral deficits caused by Aβ exposure in transgenic AD mice, was reduced in the absence of tau. This might explain the absence of electrophysiological changes upon Aβ treatment in BFCNs in this study, as tauopathy did not exist in BFCNs.

In conclusion, by comparing the pathological abnormalities of patient-specific BFCNs and cortical neurons, we revealed the diversity in pathological features among different neuronal subtypes. It might be helpful for further discovering the molecular mechanisms and identifying new therapeutic strategies for Alzheimer’s disease.

## Materials and methods

### Generation and culture of iPSCs

Generation of human iPSCs from peripheral blood mononuclear cells (PBMNCs) was performed following a previously reported protocol (Dowey et al. 2012). Mononuclear cells were isolated from the peripheral blood of adult donors by density-based centrifugal separation and cultured for 8~10 days in selective medium. The proliferated MNCs were transduced with the EBNA1/OriP-based episomal vectors EV SFFV-OS, EV SFFV-MK, and EV SFFV-BCL-XL (kind gifts from Xiao-bing Zhang, Loma Linda University, USA). The iPSC clones were isolated 2~3 weeks after transduction with KSR medium supplemented with 5 ng/ml bFGF (Pufei) and cocultured with mouse embryonic fibroblasts (MEFs) inactivated by mitomycin C or irradiation.

### Differentiation of human iPSCs

The protocol for human iPSC differentiation into cortical glutamatergic neurons was previously described (Tao et al. 2020). Briefly, iPSCs were dissociated into clumps to form EBs. Six days later, EBs were transferred into matrigel-coated culture dishes to form rosettes. At day 16, the rosettes were digested and cultured in petri-dish for 10 days to allow the formation of neural spheres. At day 26, neural spheres were digested into single cells and reseeded on matrigel-coated dishes for further neuronal differentiation and maturation.

For differentiation of iPSC to BFCN, feeder cells were first removed from the cultures by short-term digestion with 0.5 mg/ml collagenase IV. The iPSC colonies were digested into clumps by 2 mg/ml collagenase IV and cultured in nontreated petri dishes with KSR (day 0~4) and N2 (day 4~6) media supplemented with 10 μM SB431542 (Selleck) and 2 μM dorsomorphin (Sigma‒Aldrich). On day 6 after differentiation, suspended embryoid bodies (EBs) were plated onto Matrigel-coated culture plates, after which the cells formed neural tube-like rosettes. Ten days later, the NSCs/NPCs of the rosettes were dissociated and cultured in Petri dishes suspended for another 10 days to allow the formation of neural spheres. From day 6 to 26, the rosettes and spheres were maintained in N2 or N2B27 medium supplemented with SHH (R&D, 20 ng/ml) and SAG (Calbiochem, 500 nM) to induce ventralization of the NSCs/NPCs. On day 26, the neural spheres were dissociated into single cells and plated on Matrigel-coated dishes in B27 medium supplemented with neurotrophins for further neuronal differentiation and maturation. In the rosette, sphere and neuron stages, 10 ng/ml BMP9 (PeproTech) was supplemented with the corresponding media on day 10. All reagents were purchased from Life Technology if not otherwise specified.

### Measure of acetylcholine secretion

Media for iPSC-derived BFCN cultures was changed equally on day 48. After 48 h, the conditioned medium was collected. At the same time, the neurons were lysed with cell extraction buffer (Thermo Fisher Scientific) and collected. Concentration of acetylcholine in the conditioned medium were detected with the Amplex Red Acetylcholine/Acetylcholinesterase Assay Kit (Invitrogen). Total protein level of each total neuronal lysate was measured with the BCA protein assay kit (Beyotime). The quantity of acetylcholine of each culture was normalized to total protein levels correspondingly.

### RNA-seq and analysis

Two cell lines of Ctrl-, SAD- and FAD-specific iPSCs were randomly chosen for RNA sequencing and analysis. Cell samples were collected and lysed with Trizol reagent (Pufei) at day 0, 6, 16, 26, 34 and 50 during iPSC differentiation. Total RNA was extracted and RNA-seq libraries were prepared following a previously published method (Chen et al. 2017). All sample libraries were sequenced on HiSeq2500 instrument (Illumina). RNA-seq reads were aligned to UCSC hg19 reference genome using STAR (2.7.11b) with parameters –clip5pNbases 15,15 –clip3pNbases 8,8–outSAMattrIHstart 0 –outSAMmultNmax 1 –outFilterMultimapNmax 1. After removing duplicated reads, the gene count table was obtained with featureCounts (v2.0.6 using “ignoreDup” option). The raw counts were normalized with DESeq2 and the heatmap were drawn with scaled and centered log-transformed normalized count table.

### Whole-cell patch-clamp recordings

Whole-cell patch-clamp recordings were performed on iPSC-derived BFCNs using Multiclamp 700B Amplifier. The resistance of the recording micropipettes was 5-7MΩ. Pipette solution contained 143 mM K-gluconate, 3 mM KCl, 2 mM MgCl2, 10 mM HEPES, 2 mM Na2ATP and 0.025 mM BAPTA, pH 7.25~7.30. Cells were maintained in external solution at room temperature. External solution contained 150 mM NaCl, 5 mM KCl, 1 mM MgCl2, 2 mM CaCl2, 10 mM D-Glucose and 10 mM HEPES, pH 7.4. Action potentials were recorded under the current-clamp configuration. Cells were depolarized by injecting step currents to elicit action potential. To record spontaneous synaptic currents, cells were held at -70 mV and recorded in voltage-clamp mode. Data were analyzed by Clampfit and origin8.

### ELISA analysis of secreted Aβ42 and Aβ40 in iPSC-induced neurons

The culture media used for iPSC-derived cortical glutamatergic neurons or BFCNs were changed equally on day 48. After 48 h, the conditioned medium was collected. At the same time, the neurons were lysed with cell extraction buffer (Thermo Fisher Scientific) and collected. Following the instructions of manufacturer, Concentrations of Aβ42 and Aβ40 in culture medium were analyzed using Aβ42 Human Ultrasensitive ELISA Kit (Invitrogen) and Aβ40 Human ELISA Kit (Invitrogen). The total protein concentration of each total neuronal lysate was measured with a BCA protein assay kit (Beyotime). The results of both the ELISA and BCA assays were within the linear range of the standard curves. Aβ concentrations were normalized to total protein levels correspondingly.

### Preparation of Aβ oligomers

Soluble Aβ42 oligomer (AβO) solutions were prepared following a previously reported protocol (Stine et al., 2003). Briefly, synthesized human Aβ_1-42_ (LifeTein) was dissolved at a concentration of 1 mM in cold 1,1,1,3,3,3-hexafluoro-2-propanol (HFIP). The peptide was incubated at room temperature for 1 h to ensure that the peptide was monomeric and unstructured. HFIP was dried in a vacuum desiccator, and the resulting peptide film was stored at − 80 °C until use. To form oligomers, the Aβ film was dissolved in DMSO and further diluted with HEPES buffer. The sample was then centrifuged at 15,000 × g for 10 min at 4~8 °C, after which the soluble oligomers remained in the supernatant.

### Immunofluorescence staining

Immunofluorescence staining was performed to detect pluripotency and neuronal markers. The cells were fixed with 4% paraformaldehyde for 1 h at room temperature and rinsed with PBS. The fixed cells were permeabilized and blocked with PBS containing 5% BSA and 0.3% Triton X-100 for 1 h at room temperature. Then, the cell cultures were incubated overnight at 4 ℃ and supplemented with primary antibodies. The secondary antibodies were then applied for 1.5 h at room temperature. The primary antibodies used in this study: anti-OCT4 (1:200; Santa Cruz, SC-5279), anti-TRA-1–60 (1:50; Millipore, SCR001), anti-TRA-1–81 (1:50; Millipore, SCR001), anti-SSEA4 (1:50; Millipore, SCR001), anti-TUJ1 (1:1000; Covance, MMS-435P, MRB-435P), anti-MAP2 (1:200; Sigma, M4403), anti-NEUN (1:200; Millipore, ABN78), anti-ChAT (1:100, Millipore, AB144P), anti-VAChT (1:200, Synaptic Systems, 139,013), anti-ISL1 (1:200, DSHB, 40.2D6),anti-vGLUT (1:300; Synaptic Systems, 135,302), anti-GAD67 (1:300; Millipore, MAB5406), anti-TH (1:300; Millipore, AB152), anti-HB9 (1:200, DSHB, 81.5C10), anti-Gbx1 (1:100, DSHB, AB_2618646) or anti-P75NTR (1:100, Millipore, AB1554). Images were captured on an Olympus IX71, FV3000 or Leica TCS SP5 confocal laser microscope.

### Western blotting

Total proteins were extracted from neurons using cell extraction buffer (Invitrogen, FNN0011). The protein samples were resolved via SDS‒PAGE, transferred to PVDF membranes and blotted with antibodies. The primary antibodies used in this study were anti-pTau Ser202/Thr205 (Thermo), anti-pTau Ser396 (Abcam), anti-pTau Thr231 (Abcam), anti-Tau (Abcam), anti-Caspase 3 (Santa Cruz), and anti-PARP (Cell Signaling), anti- BACE1 (Cell signaling technology), anti-ADAM10 (Abcam), anti-6E10 (Biolegend). All the experiments were performed with two to three biological replications and duplicate samples for each iPSC line.

### Microelectrode array recording

Sterile CytoView 24-well MEA plates (Axion BioSystems) were coated with freshly made 0.1% polyethyleneimine (PEI) (Sigma, P3143) solution for 1 h at 37 ℃. After aspiration of the PEI solution, the wells were rinsed with sterile water 4 times and air-dried overnight in a tissue culture hood. Neural spheres derived from iPSCs on day 26 of differentiation were dissociated into single cells with accutase, suspended in B27 medium containing 10 μg/ml laminin and then replated on MEA plates at a density of 200,000 cells/well. On the second day, 1000 astrocytes were plated in each well and cocultured with iPSC-derived neurons. MEA recordings of spontaneous activities were performed using the Maestro system of Axion Biosystems. The extracellular voltage was recorded at a sampling rate of 12.5 kHz. Spikes were identified using a detection threshold set to ± 6 times the standard deviation of the baseline electrode noise, and bursts were identified with a minimum of five spikes on a single electrode and a maximum interspike interval of 0.1 s. The Neural Metrics Tool (Axion BioSystems) was used for further analysis of the raw data. At least 3 independent recordings were performed for each experiment.

### Statistics

For ELISA analysis, Aβ concentrations were normalized to total protein levels in whole-cell lysates and relative to the mean of the control. For western blot analysis, the data sets were normalized to the expression of GAPDH and relative to the mean of the control. All the statistical analyses were performed using GraphPad Prism. Sample size (*n*) values are provided in the relevant text, figures and figure legends. The data for each sample were calculated from two to three technical replications. The statistical analyses were performed on data from three independent experiments. All the data are presented as the mean ± SD. Two-tailed Student’s t tests were used for two groups, and one-way ANOVA with the Newman‒Keuls post hoc test was used for more than two groups. *P* < 0.05 was considered to indicate statistical significance. For all, * *P* < 0.05, ** *P* < 0.01 and *** *P* < 0.001.

## Supplementary information

Supplementary Material 1: Fig. S1. Characterization of human iPSC-induced neurons and comparison of the differentiation capacities of control, SAD and FAD patient-specific iPSCs into BFCNs. Fig. S2. BFCNs express less APP than cortical neurons. Fig. S3. Cortical neurons and BFCNs exhibit different tau phosphorylation responses to toxic AβO. Fig. S4. Extracellular voltage traces confirmed the AβO-derived decrease in electrical activity in cortical neurons. Table S1. Summary of donor information

## Data Availability

All RNA sequencing data are available at the China National Center for Bioinformation (CNCB) under accession number HRA008415.

## Abbreviations

AD: Alzheimer’s disease
BFCN: Basal forebrain cholinergic neuron
AβO: Aβ42 oligomer
SAD: Sporadic AD
FAD: Familial AD
Aβ: β-Amyloid peptide
ChAT: Choline acetyltransferase
AChE: Acetylcholinesterase
iPSC: Induced pluripotent stem cell
PBMNC: Peripheral blood mononuclear cell
SHH: Sonic hedgehog
SAG: Smoothened agonist
NSC: Neural stem cell
NPC: Neural progenitor cell
AP: Action potential
PSC: Post-synaptic current
APP: Amyloid precursor protein
MEA: Multielectrode array
TTX: Tetrodotoxin
GWAS: Genome-wide association study
MEF: Mouse embryonic fibroblast
EB: Embryoid bodie
HFIP: 1,1,1,3,3,3-Hexafluoro-2-propanol
PEI: Polyethyleneimine
