# Supplementary material for: Subtype-specific neurons from patient iPSCs display distinct neuropathological features of Alzheimer’s disease
Source: Cell Regen. 2024 Oct 10;13:21. doi: 10.1186/s13619-024-00204-y (PMC11467140; doi:10.1186/s13619-024-00204-y)
Supplement: Supplementary file 1 — Supplementary Material 1: Fig. S1. Characterization of human iPSC-induced neurons and comparison of the differentiation capacities of control, SAD and FAD patient-specific iPSCs into BFCNs. Fig. S2. BFCNs express less APP than cortical neurons. Fig. S3. Cortical neurons and BFCNs exhibit different tau phosphorylation responses to toxic AβO. Fig. S4. Extracellular voltage traces confirmed the AβO-derived decrease in electrical activity in cortical neurons. Table S1. Summary of donor information [file 13619_2024_204_MOESM1_ESM.docx]

**Fig. S1. Characterization of human iPSC-induced neurons and comparison of the differentiation capacities of control, SAD and FAD patient-specific iPSCs into BFCNs.**

1. Immunofluorescence images of human iPSC-derived cells expressing the neuronal markers TUJ1 (a) and MAP2 (b), the BFCN marker GBX1 (c) and P75NTR (d), the glutamatergic neuronal marker vGLUT (e), the GABAergic neuronal marker GAD67 (f), the dopaminergic neuronal marker TH (g) and the spinal cord motor neuronal marker HB9 (h) at differentiation day 50. Arrows, representative neurons with positive staining. Scale bars, 50 μm.
2. Percentages of human iPSC-derived BFCNs firing no action potential (AP), one AP and repetitive AP at differentiation days 55-65. n=12.
3. Representative traces of spontaneous AP recorded in human iPSC-derived BFCNs at days 55-65.
4. Representative traces showing spontaneous PSCs received by human iPSC-derived BFCNs at days 55-65.
5. Representative immunofluorescence images of human iPSC-derived cortical neurons expressing cortical marker TBR1 (a), mature neuronal marker NEUN (b) and glutamatergic neuronal marker vGLUT (c) at differentiation day 50. Scale bars, 50 μm.
6. Quantification of the percentage of cortical neurons induced from human iPSCs shown in Fig. S1E. n = 3. Data are presented as the means ± SDs.

**Fig. S2. BFCNs express less APP than cortical neurons.**

1. The protein levels of APP, BACE1 and ADAM10 expressed in control-, SAD- and FAD-iPSC derived cortical neurons and BFCNs were analyzed via western blotting.
2. Quantification of the related protein levels detected by western blotting shown in Fig. S2A. The data are presented as the means ± SDs. *P<0.05, **P<0.01 and ***P<0.001.

**Fig. S3. Cortical neurons and BFCNs exhibit different tau phosphorylation responses to toxic AβO.**

1. Control iPSC-derived cortical neurons and BFCNs were treated with synthesized human Aβ1-42 oligomers at different concentrations ranging from 25 to 300 μg/ml. After 48 hours, the levels of pTau 231 and total tau were analyzed via western blotting. Human Aβ42-1 oligomers were used as scramble negative controls.
2. The quantification of the related protein levels was performed via western blotting, as shown in Fig. S3A. The data are presented as the means ± SDs. *P<0.05, **P<0.01 and ***P<0.001.
3. Control-, SAD- and FAD-iPSC-derived cortical neurons and BFCNs were treated with synthesized human Aβ1-42 (Aβ42) oligomers at a concentration of 200 μg/ml. After 48 hours, the levels of pTau 396 and total tau were analyzed via western blotting.
4. The quantification of the related protein levels was performed via western blotting, as shown in Fig. S3C. The data are presented as the means ± SDs. *P<0.05, **P<0.01 and ***P<0.001.

**Fig. S4. Extracellular voltage traces confirmed the** **AβO-derived decrease in electrical activity in cortical neurons.**

Representative extracellular voltage traces for 60 seconds from a single electrode showing changes in spontaneous activity before or 2 days after AβO treatment. The red lines indicate ±0.01 mV.

**Supplementary Table S1. Summary of donor information**

| **Donor Code** | **Diagnosis** | **Gender** | **Age** | **Genotype** |
| --- | --- | --- | --- | --- |
| 1 | Healthy | M | 59 | - |
| Control-2 | Healthy | M | 70 | - |
| Control-3 | Healthy | F | 68 | - |
| SAD03 | SAD | M | 62 | - |
| SAD04 | SAD | M | 55 | - |
| SAD05 | SAD | M | 57 | - |
| FAD01 | FAD | F | 48 | PSEN1 c. 604A>T,  p. I202F |
| FAD02 | FAD | M | 36 | APP c. 2149G>A,  p. V717I |
